# Association between helicobacter pylori infection and subclinical atherosclerosis: A systematic review and meta-analysis

**DOI:** 10.1097/MD.0000000000027840

**Published:** 2021-11-19

**Authors:** Xianghong Wang, Qian He, Donghua Jin, Baohua Ma, Kecheng Yao, Xiulan Zou

**Affiliations:** a Department of Endocrinology, The Third Clinical Medical College of China Three Gorges University/Gezhouba Central Hospital of Sinopharm, Yichang, Hubei Province, China; b Department of Geriatrics, The People's Hospital of China Three Gorges University/The First People's Hospital of Yichang, Yichang, Hubei Province, China; c Department of Neurosurgery, The Second Affiliated Hospital of Zhengzhou University, Zhengzhou, Henan Province, China; d Department of Endocrinology, The People's Hospital of China Three Gorges University/The First People's Hospital of Yichang, Yichang, Hubei Province, China; e Healthcare Center, The People's Hospital of China Three Gorges University/The First People's Hospital of Yichang, Yichang, Hubei Province, China.

**Keywords:** carotid intima-media thickness, helicobacter pylori, meta-analysis, pulse wave velocity, subclinical atherosclerosis

## Abstract

**Background:**

The relationship between Helicobacter pylori (*H. pylori*) infection and subclinical atherosclerosis has been confirmed, but these conclusions are still controversial. Therefore, we have performed a systematic review and meta-analysis to assess the association between *H. pylori* infection and subclinical atherosclerosis.

**Methods:**

Databases including PubMed, Embase, Web of Science were searched for the articles on the association of carotid intima-media thickness or pulse wave velocity with *H. pylori* infection published up to January 1, 2020. Stata 12.0 was used to calculate standardized mean difference (SMD) and 95% confidence interval (95% CI); the *I*^*2*^ test was used to evaluate heterogeneity between studies and sensitivity analysis and subgroup analysis were used to explore the source of heterogeneity. Funnel plot, Begg test, and Egger test were used to estimate publication bias.

**Results:**

Data were extracted from 18 studies involving 6776 subjects with *H. pylori* positive and 7794 with *H. pylori* negative. *H. pylori* positive subjects is significantly associated with increased subclinical atherosclerosis as determined by carotid intima-media thickness (SMD: 0.376 mm; 95% CI: 0.178, 0.574; *P* < .001, I^2^ = 90.6%), pulse wave velocity (SMD: 0.320 m/s; 95% CI: 0.242, 0.398; *P* < .001, I^2^ = 52.6%), compared with *H. pylori* negative. Similar results were observed when subgroups analysis were stratified according to age, male ratio, geographical location, *H. pylori* diagnosis, and study design. Sensitivity analyses showed that our results were robust. The Begg test or Egger test showed no significant publication bias (all *P* > .05).

**Conclusions:**

This meta-analysis confirmed a significant association between *H. pylori* and subclinical atherosclerosis, which will help *H. pylori* patients to establish effective strategies for the prevention and control of cardiovascular events.

## 1 Introduction

Helicobacter pylori (*H*. *pylori)* is a gram-negative spiral bacterium that is found on the surface of the gastric epithelium. More than 50% of the world population is infected with *H*. *pylori*, which is one of the most common chronic infections in the world.^[1]^ H. *pylori* infection is related to peptic ulcers, chronic gastritis, mucosa-associated lymphoid tissue lymphoma, and gastric cancer.^[2,3]^ Growing evidence has also supported a role for *H*. *pylori* infection in the development of gastrointestinal diseases, including cardiovascular diseases (CVDs) and type 2 diabetes.^[4–7]^

Atherosclerosis is an important risk factor for CVDs. Since Patel et al^[8]^ first found that *H. pylori* infection was independently associated with coronary heart disease in the 1990s, an increasing number of studies have focused on the influence of *H. pylori* infection on the development of coronary heart disease. However, subclinical atherosclerosis is a precursor symptom that can develop into clinical coronary heart disease. With the improvement of CVD diagnosis methods, carotid intima-media thickness (CIMT) and pulse wave velocity (PWV) have improved the ability to detect subclinical atherosclerosis. CIMT and PWV have been widely used to evaluate subclinical atherosclerosis,^[9–11]^ which is closely related to cardiovascular events.^[12,13]^ CIMT reflects the early structural changes related to arterial wall ageing,^[14]^ and PWV refers to the PWV in the artery, which is used to evaluate the degree of arterial stiffness.^[15]^ A meta-analysis showed that every 0.1 mm increase in CIMT increased the risk of myocardial infarction by 15% and the risk of stroke by 17%.^[16,17]^ In the Munakata^[18]^ study, the correlation between PWV and the risk of CVD was persistent, and an increase in PWV by 20% was associated with a 1.3-fold increase in the risk of cardiovascular events. CIMT and PWV, as markers of subclinical atherosclerosis, have been widely studied in terms of the influence of *H. pylori* infection on the arterial vascular system. Therefore, it is very important to evaluate the relationship between *H. pylori* infection and subclinical atherosclerosis.

In the past few years, several studies have evaluated the relationship between *H. pylori* infection and subclinical atherosclerosis. Some studies have shown that there is a significant statistical correlation between *H. pylori* infection and subclinical atherosclerosis.^[6,19]^ However, other studies have shown no significant association between subclinical atherosclerosis and *H. pylori* infection.^[20,21]^ In view of the controversial published literature, whether subclinical atherosclerosis is truly associated with *H. pylori* infection remains unclear. There are no previous meta-analyses that assessed the relationship between subclinical atherosclerosis and *H. pylori* infection. Therefore, we conducted a systematic review and meta-analysis of all available evidence to date on the relationship between *H. pylori* infection and subclinical atherosclerosis (CIMT, PWV).

### 1.1 Search strategy

According to the PRISMA guidelines,^[22]^ we conducted a systematic literature search in PubMed, EMBASE, and Web of Science to identify all observational studies published through January 1, 2020 that examined the association between *H. pylori* and subclinical atherosclerosis (CIMT, PWV). We used the following search terms in titles and abstracts: (‘Helicobacter pylori’ OR ‘H. pylori’ OR ‘Helicobacter’ OR ‘Campylobacter pylori’ OR ‘helicobacter infections’) AND (‘carotid intima-media thickness’ OR ‘intima-media thickness’ OR ‘carotid artery internal’ OR ‘pulse wave velocity’ OR ‘arterial stiffness’ OR ‘subclinical atherosclerosis’). The literature was limited to only articles published in English.

### 1.2 Inclusion criteria

The inclusion criteria were as follows: Observational studies; The *H. pylori* positive patients were the experimental group and the *H. pylori* negative subjects were the control group; Reported the differences in CIMT/PWV between *H. pylori* positive patients and *H. pylori* negative subjects.

### 1.3 Exclusion criteria

The exclusion criteria were as follows: Commentaries, meta-analysis, animal studies, review, or case report; Duplicated or repeated study or with great similarity in the sample or content with another study; The study did not provide the mean or standard deviation of CIMT/PWV.

### 1.4 Data extraction and quality assessment

Two reviewers (Xianghong Wang and Kecheng Yao) independently extracted the following data and assessed the quality of each study. For each pooled article, we collected the name of the first author, date of publication, study design, study country, methods of *H. pylori* detection, age, male ratio, number of participants, and the value of CIMT or PWV. The quality of the studies was assessed by the Newcastle–Ottawa Scale (NOS),^[23]^ which is a scale designed to assess the risk of bias of non-randomized studies in meta-analyses. The NOS scoring system includes 3 aspects of the study: selection, comparability, exposure or outcome, and a score ranging from 0 to 9 is given. The higher the score, the better the quality of the methodology. The quality evaluation results of the NOS are reported in Table 1. In this meta-analysis, if the data were repeated in different publications, we chose the most informative full text. Any discrepancies between the reviewers in research selection, quality assessment, or data extraction were addressed by re-evaluating the original with a third author (He Qian).

**Table 1:** Characteristics of literatures included in the meta-analysis.

Study and year	Country	Region	Participants (Hp+/Hp–)	Age(Hp+/Hp–)	Male ratio (%) (Hp+/Hp–)	Diagnosis of Hp infection	Study type	Variables (Hp+/Hp–)	NOS score
CIMT (mm)									
Zhang (male) 2019 (a)	China	Asia	659/945	≤50	100.0/100.0	UBT	Cross-sectional	0.69 ± 0.08/0.67 ± 0.09	7
Zhang (male) 2019 (b)	China	Asia	770/1436	>50	100.0/100.0	UBT	Cross-sectional	0.76 ± 0.12/0.76 ± 0.11	7
Zhang (female) 2019	China	Asia	668/1301	∗	0.0/0.0	UBT	Cross-sectional	0.66 ± 0.09/0.67 ± 0.11	7
Karadag 2018	Turkey	Europe	69/21	49.5 ± 7.5/52.0 ± 7.9	47.8/42.9	B	Cross-sectional	0.76 ± 0.10/0.67 ± 0.08	9
Feng 2018	China	Asia	42/49	46.64 ± 0.54/46.61 ± 0.53	78.6/77.6	UBT	Cross-sectional	0.75 ± 0.011/0.75 ± 0.009	6
Judaki 2017	Iran	Asia	40/40	45.64 ± 8.32/46.52 ± 5.52	45.0/57.5	UBT, B, cultivation	Case-control	0.58 ± 0.13/0.48 ± 0.32	6
Bao-Ge 2017	China	Asia	35/47	46.5 ± 7.02/46.7 ± 6.8	91.4/87.2	UBT	Cross-sectional	0.71 ± 0.19/0.70 ± 0.16	8
Xu 2016	China	Asia	208/156	63.2 ± 10.4/62.8 ± 11.7	53.8/51.9	UBT	Cross-sectional	1.12 ± 0.18/0.93 ± 0.15	7
Mete 2013	Turkey	Europe	103/31	49.8 ± 8.7/50.2 ± 9.3	42.7/37	B	Cross-sectional	0.73 ± 0.17/0.57 ± 0.07	7
Akbas 2010	Turkey	Europe	30/31	40.9 ± 10.3/ 42.3 ± 9.4	43.3/45.2	UBT or SAT	Case-control	0.71 ± 0.10/0.65 ± 0.06	6
Hamed 2008	Egypt	Africa	68/12	47.6 ± 9.1/48.2 ± 9.3	51.5/33.3	S	Case-control	0.84 ± 0.17/0.78 ± 0.1	8
Koksal 2004	Turkey	Europe	63/21	46.7 ± 14.7/45.1 ± 7.1	27.0/33.3	S	Cross-sectional	0.80 ± 0.30/0.80 ± 0.30	7
Diomedi 2004	Italy	Europe	146/39	67.8 ± 11.8/66.9 ± 15.8	53.8/67.1	S	Cross-sectional	1.06 ± 0.23/1.01 ± 0.17	6
PWV (m/s)									
Lee 2018	Korea	Asia	224/239	53.2 ± 7.9/55.3 ± 8.9	76.3/69.0	B	Cross-sectional	14.25 ± 0.14/14.20 ± 0.13	9
Torisu 2009	Japan	Asia	210/133	61.82 ± 2.47/61.61 ± 2.41	44.0/41.0	S	Cross-sectional	16.12 ± 3.95/15.67 ± 3.22	7
Ohnishi 2008	Japan	Asia	70/60	66.7 ± 11.3/60.0 ± 12.2	57.1/55.0	S	Cross-sectional	18.77 ± 5.5/15.85 ± 3.31	9
Honda 2008	Japan	Asia	166/92	50.4 ± 7.8/47.6 ± 7.0	72.3/68.5	S	Cross-sectional	7.77 ± 0.17/7.38 ± 0.22	7
Yoshikawa 2007	Japan	Asia	668/279	54.2 ± 8.8/50.2 ± 8.9	60.6/54.8	S	Cross-sectional	14.56 ± 2.68/13.75 ± 2.55	8
Saijo (male) 2005	Japan	Asia	1586/1826	50.3 ± 6.1/46.7 ± 7.0	100.0/100.0	S	Cross-sectional	13.97 ± 2.10/13.44 ± 1.87	8
Saijo (female) 2005	Japan	Asia	358/496	48.7 ± 6.8/45.4 ± 7.1	0.0/0.0	S	Cross-sectional	12.73 ± 1.84/12.33 ± 1.76	8
Adachi 2003	Japan	Asia	573/423	50.9 ± 0.4/46.1 ± 0.4	70.0/67.0	S	Cross-sectional	7.74 ± 0.08/7.71 ± 0.09	7

B = biopsy or histology, CIMT = carotid intima-media thickness, HP = helicobacter pylori, NOS = Newcastle–Ottawa Scale, PWV = pulse wave velocity, S = serology, SAT = stool antigen test, UBT = urea breath test. ^∗^: not reported.

### 1.5 Statistical analysis

All statistical analyses were conducted using STATA version 12.0 software (StataCorp, College Station, TX). Based on the heterogeneity of the articles, fixed-effects or random-effects models were used to calculate the pooled standardized mean difference (SMD) and 95% confidence intervals (CIs). Heterogeneity was assessed by the Q test and I^2^ statistic.^[24]^ If the I^2^ statistic was ≥50%, a random-effects model was applied. If the I^2^ statistic was <50%, a fixed-effects model was used. We performed a subgroup analysis based on the mean age of the *H. pylori* patients (>50 years or ≤50 years), geographical region (Asia or Europe), male ratio (>50% or ≤50%), study design (case-control or cross-sectional), and diagnostic method of *H. pylori* (invasive or non-invasive). At the same time, to evaluate the reliability of the meta-analysis, sensitivity analysis was applied to examine the impact of individual studies on the overall merger effect. Furthermore, a visual funnel plot was utilized to evaluate publication bias qualitatively, and Egger^[25]^ tests and Begg tests^[26]^ were used to quantitatively detect the risk of publication bias. A *P* value <.05 was considered statistically significant.

## 2 Results

### 2.1 Literature search and study characteristics

**Figure 1:**
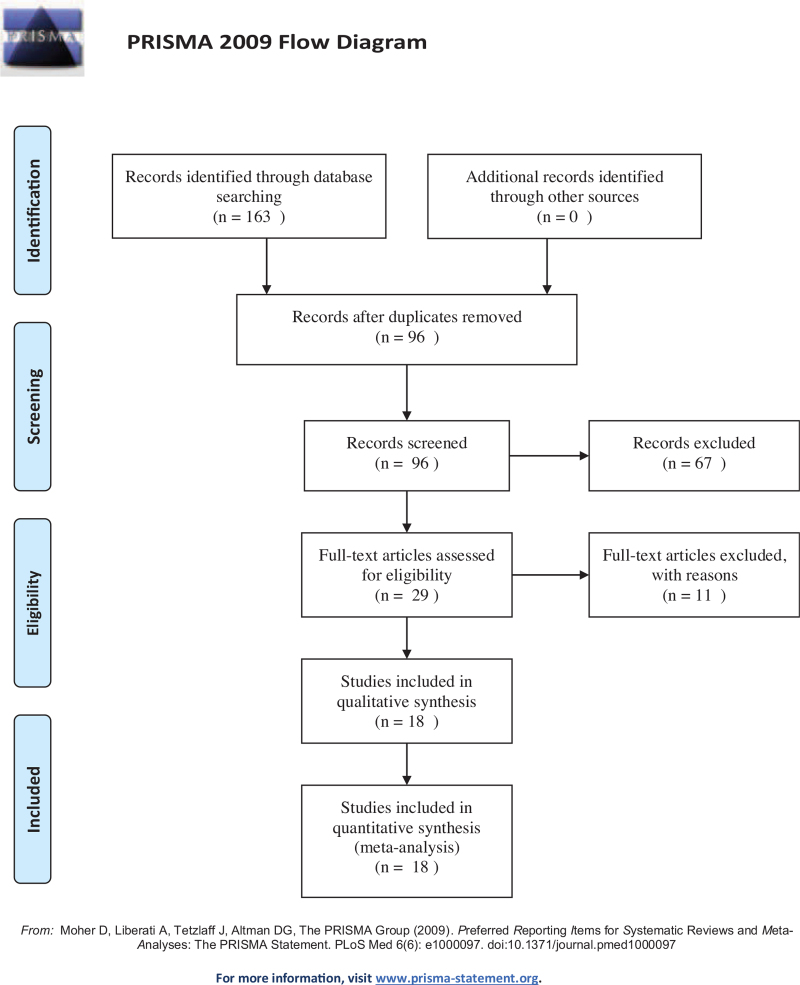
Flowchart of the study selection.

Through the search strategy, 163 articles were initially identified. After deleting repetitive articles, screening titles and abstracts, and reading the full text, 18 articles^[19,20,27–42]^ were included in this meta-analysis (Fig. 1). The major characteristics of the included studies are presented in Table 1. The meta-analysis involved 14,570 subjects, including 6776 patients with *H. pylori* infection and 7794 controls without *H. pylori* infection. Eleven studies^[20,27–36]^ had data on CIMT values, and 7 studies^[19,37–42]^ reported PWV values. Five of the studies^[27,32,33,35,36]^ were from Europe, 12 studies^[20,28–31,34,37–42]^ were from Asia, and 1^[34]^ was from Africa. These studies were published between 2004 and 2019. Among them, 2 articles^[20,40]^ reported information on male and female *H. pylori*-positive and *H. pylori*-negative controls. Our meta-analysis contained 3 case-control studies^[29,33,34]^ and 15 cross-sectional studies.^[19,20,27,28,30–32,35–42]^ The mean age of the participants ranged from 40.9 to 67.8 years, and the percentage of men ranged from 0% to 100%. Regarding the *H. pylori* testing method, 4 studies^[20,28,30,31]^ used the urea breath test, 9 studies^[34–42]^ used serology to detect antibodies to *H. pylori*, 3 studies^[19,27,32]^ used biopsy or histology methods, and 2 studies^[29,33]^ used multiple methods. The quality of the included studies ranged from 6 to 9.

### 2.2 Meta-analysis

#### 2.2.1 CIMT between patients with *H. pylori* infection and controls without *H. pylori* infection

**Figure 2:**
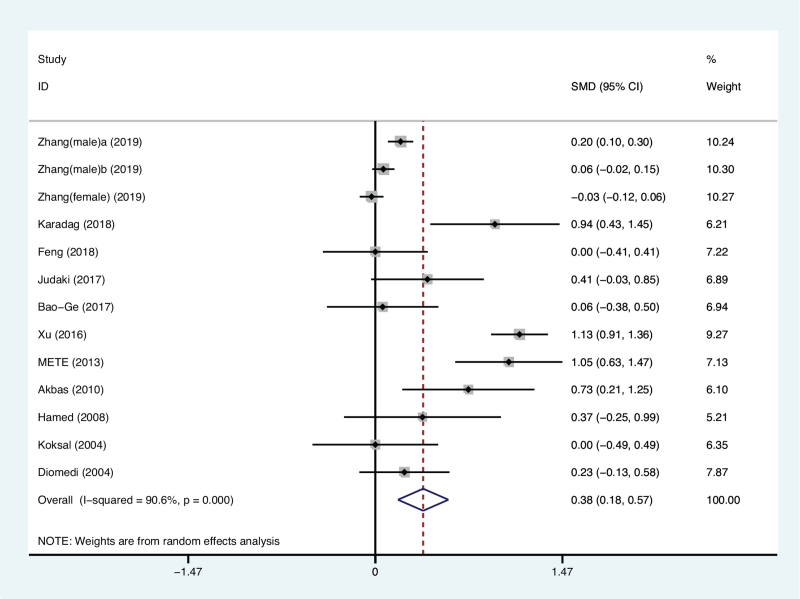
Forest plot for carotid intima-media thickness in patients with *H. pylori* positive and *H. pylori* negative controls. CI = confidence interval, SMD = standardized mean difference.

A total of 13 studies involving 7030 participants estimated the relationship between *H. pylori* infection and CIMT, including 2901 patients *with H. pylori* infections and 4129 controls without *H. pylori* infections. Our results revealed that compared with the group without *H. pylori* infection, the CIMT of patients with *H. pylori* infection was significantly thicker (SMD: 0.376 mm; 95% CI: 0.178, 0.574; *P* < .001, Fig. 2). There was significant heterogeneity by I^2^ = 90.6%, *P* < .001, so a random-effects model was used.

#### 2.2.2 PWV between patients with *H. pylori* infection and controls without *H. pylori* infection

**Figure 3:**
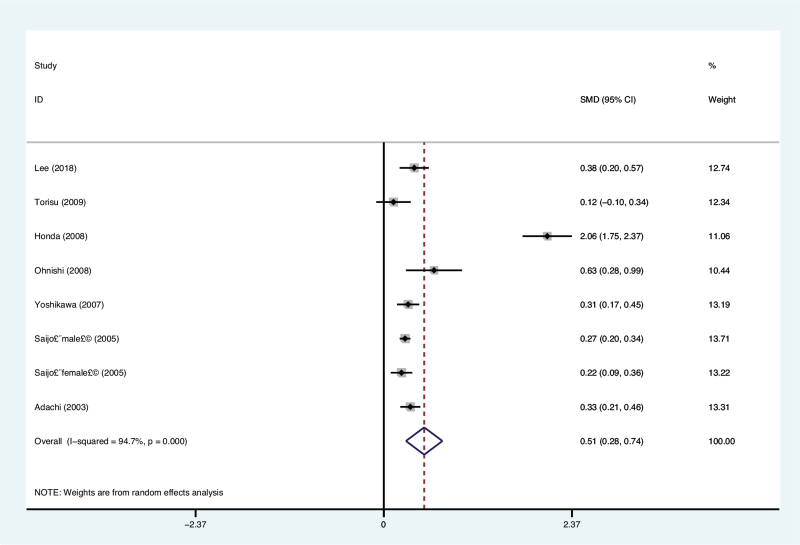
Forest plot for pulse wave velocity in patients with *H. pylori* positive and *H. pylori* negative controls. CI = confidence interval, SMD = standardized mean difference.

A total of 8 studies involving 7540 participants estimated the relationship between *H. pylori* infection and PWV, including 3875 patients with *H. pylori* infection and 3665 controls without *H. pylori* infection. Patients with *H. pylori* infection demonstrated significantly higher PWV than controls without *H. pylori* infection (SMD: 0.320 m/s; 95% CI: 0.242, 0.398; *P* < .001, Fig. 3), yet significant between-study heterogeneity was observed (I^2^ = 52.6%, *P* < .001), so a random-effects model was used.

### 2.3 Subgroup analysis

#### 2.3.1 Subgroup analysis for CIMT in *H. pylori* infection

The association between *H. pylori* infection and CIMT tended to be more pronounced in the subgroups of mean age >50 years (SMD: 0.671 mm; 95% CI: 0.105, 1.237; *P* = .02), male ratio less than 50% (SMD: 0.481 mm; 95% CI: 0.086, 0.876; *P* = .017), study performed in Europe (SMD: 0.584 mm; 95% CI: 0.179, 0.989; *P* = .005), *H. pylori* infection evaluation by invasive testing (SMD: 0.797 mm; 95% CI: 0.397, 1.197; *P* < .001), and the study type was a case-control study (SMD: 0.505 mm; 95% CI: 0.209, 0.800; *P* = .001), which showed a significant test for interaction. These results indicate that the above factors are responsible for the high heterogeneity in the overall analysis to a certain extent. Table 2 summarizes the detailed results of the grouping analysis.

**Table 2:** Subgroup analysis for carotid intima-media thickness in *H. pylori* infection.

Variables	Group	No. of studies	No. of patients	SMD (95%CI)	*P*	I^2^ (%)	*P*_hetero_
Total		13	7030	0.376 (0.178, 0.574)	<.001	90.6	<.001
Age (yr)	>50	5	2979	0.671 (0.105, 1.237)	.020	95.9	<.001
	≤50	7	2083	0.213 (0.077, 0.349)	.002	15.5	.312
	Undefined	1	1969	−0.029 (−0.123, 0.064)	.536	–	–
Male (%)	>50	6	4532	0.295 (0.002, 0.588)	.048	93.6	<.001
	≤50	7	2498	0.481 (0.086, 0.876)	.017	86.9	<.001
Region	Asia	7	6396	0.263 (0.025, 0.501)	.030	93.7	.003
	Europe	5	554	0.584 (0.179, 0.989)	.005	75.1	<.001
	Africa	1	80	0.370 (−0.246, 0.987)	.239	–	–
*H. pylori* diagnosis method	Invasive	3	304	0.797 (0.397, 1.197)	<.001	56.6	.1
	Non-invasive	10	6726	0.268 (0.062, 0.474)	.011	90.9	<.001
Study design	CS	10	6809	0.347 (0.127, 0.567)	.002	92.6	<.001
	CC	3	221	0.505 (0.209, 0.800)	.001	0	.681

CC = case-control design, CI = confidence interval, CS = cross-sectional design, *P*_hetero_ = *P* value of heterogenicity, SMD = standardized mean difference.

#### 2.3.2 Subgroup analysis for PWV in *H. pylori* infection

The association between *H. pylori* infection and PWV tended to be more pronounced in the subgroups of mean age >50 years (SMD: 0.307 m/s; 95% CI: 0.231, 0.384; *P* < .001; I^2^ = 38.3%), male ratio more than 50% (SMD: 0.321 m/s; 95% CI: 0.250, 0.392; *P* < .001, I^2^ = 28.0%), and *H. pylori* infection evaluation by non-invasive testing (SMD: 0.315 m/s; 95% CI: 0.229, 0.401; *P* < .001, I^2^ = 57.6%), which showed a significant test for interaction. These results suggest that these factors may also be the source of the statistical heterogeneity. The detailed findings of the subgroup analyses are summarized in Table 3.

**Table 3:** Subgroup analysis for pulse wave velocity in *H. pylori* infection.

Variables	Group	No. of studies	No. of patients	SMD (95%CI)	*P*	I^2^ (%)	*P*_hetero_
Total		8	7403	0.509 (0.279, 0.738)	<.001	94.7	<.001
Age (yr)	>50	7	6549	0.558 (0.287, 0.828)	<.001	95.3	<.001
	≤50	1	854	0.223 (0.087, 0.359)	.001	–	–
Male (%)	>50	6	6206	0.632 (0.328, 0.937)	<.001	96.0	<.001
	≤50	2	1197	0.195 (0.079, 0.310)	.001	0	.441
*H. pylori* diagnosis method	Invasive	1	463	0.384 (0.200, 0.568)	<.001	–	–
	Non-Invasive	7	6940	0.509 (0.270, 0.790)	<.001	95.4	<.001

CI = confidence interval, CIMT = carotid intima-media thickness, *P*_hetero_ = *P* value of heterogenicity, SMD = standardized mean difference.

### 2.4 Sensitivity analysis

To assess the stability of the meta-analysis results, we performed sensitivity analysis by removing the studies one-by-one and performing an additional meta-analysis for each set. Statistically similar results were obtained after sequentially excluding each study from the analyses of CIMT and PWV, suggesting that our results are robust (Table 4).

**Table 4:** CIMT, PWV between patients with *H. pylori* positive and controls *H. pylori* negative in sensitivity analyses.

		CIMT					PWV		
Excluding literature	Year	SMD (95% CI)	*P*	I^2^ (%)	Excluding literature	Year	SMD (95% CI)	*P*	I^2^ (%)
Over all		0.376 (0.178, 0.574)	<.001	90.6	Over all		0.509 (0.279, 0.738)	<.001	94.7
Zhang (male) a	2019	0.401 (0.158, 0.644)	.001	91.3	Lee	2018	0.530 (0.270, 0.790)	<.001	95.4
Zhang (male) b	2019	0.416 (0.170, 0.661)	.001	90.9	Torisu	2009	0.565 (0.312, 0818)	<.001	95.3
Zhang (female)	2019	0.424 (0.195, 0.653)	<.001	89.6	Ohnishi	2008	0.495 (0.250, 0.739)	<.001	95.3
Karadag	2018	0.338 (0.138, 0.538)	.001	90.7	Honda	2008	0.290 (0.225, 0.355)	<.001	30.9
Feng	2018	0.406 (0.198, 0.613)	<.001	91.3	Yoshikawa	2007	0.545 (0.273, 0.817)	<.001	95.4
Judaki	2017	0.374 (0.167, 0.580)	<.001	91.3	Saijo(male)	2005	0.559 (0.247, 0.870)	<.001	95.2
Bao-Ge	2017	0.400 (0.193, 0.608)	<.001	91.4	Saijo(female)	2005	0.558 (0.287, 0.828)	<.001	95.3
Xu	2016	0.261 (0.114, 0.409)	.001	78.4	Adachi	2003	0.542 (0.264, 0.820)	<.001	95.4
Mete	2013	0.322 (0.127, 0.518)	.001	90.0					
Akbas	2010	0.353 (0.150, 0.555)	.001	91.1					
Hamed	2008	0.376 (0.172, 0.581)	<.001	91.4					
Koksal	2004	0.402 (0.195, 0.608)	<.001	91.4					
Diomedi	2004	0.389 (0.180, 0.599)	<.001	91.4					

CI = confidence interval, CIMT = carotid intima-media thickness, PWV = pulse wave velocity, SMD = standardized mean difference.

### 2.5 Publication bias

**Figure 4:**
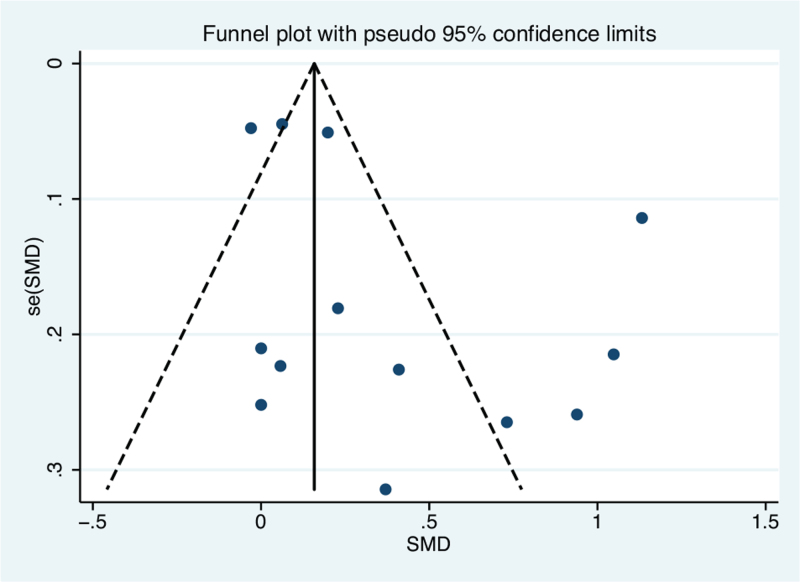
Visual evaluation of the funnel plot for the included studies on *H. pylori* positive and controls *H. pylori* negative: carotid intima-media thickness. SMD = standardized mean difference.

**Figure 5:**
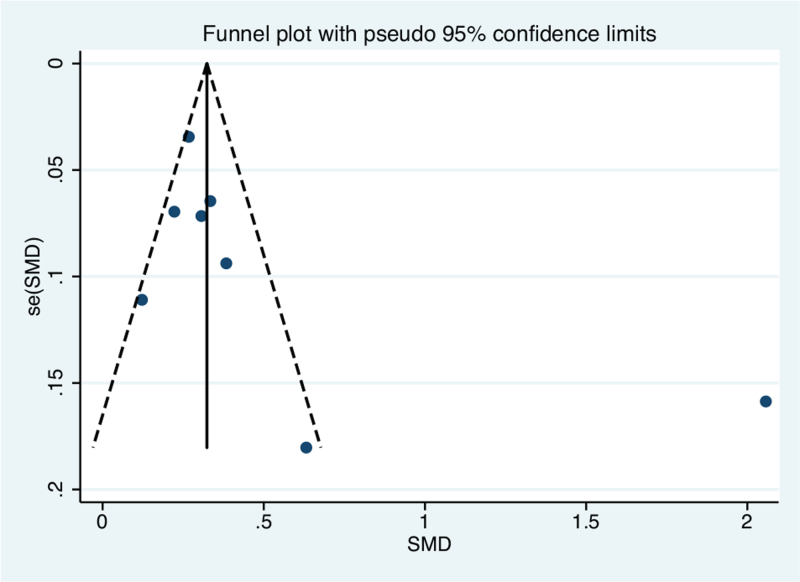
Visual evaluation of the funnel plot for the included studies on *H. pylori* positive and controls *H. pylori* negative: pulse wave velocity. SMD = standardized mean difference.

A slightly asymmetrical distribution of studies was detected among those that assessed CIMT (Fig. 4), but the results of Begg test (*P* = .300) and Egger test (*P* = .083) were both non-significant. For the evaluation of the PWV studies, the funnel plot was symmetrically distributed (Fig. 5), and Begg test (*P* = .386) and Egger test (*P* = .219) revealed that there was no significant publication bias.

## 3 Discussion

To our knowledge, this work is the first systematic review and meta-analysis evaluating the relationship between *H. pylori* infection and subclinical atherosclerosis. Our meta-analysis includes 18 observational studies demonstrating that *H. pylori* infection is associated with subclinical atherosclerosis. The CIMT was thicker and the PWV was higher in patients with *H. pylori* infection than in controls without *H. pylori* infection. Similar results were observed when the subgroup analysis was stratified by geographic location, study design, sex and age, and *H. pylori* detection methods. In addition, the sensitivity analysis demonstrated that our results were robust.

In the subgroup analysis of the relationship between geographical regions and CIMT, we explored the significant relationship between *H. pylori* infection and CIMT in Asia and Europe. However, there was still a high degree of heterogeneity in these subgroups, so we should interpret these results carefully. To determine the exact impact of geographic location on the relationship between *H. pylori* and subclinical atherosclerosis risk, the role of genetic and environmental factors should be further studied. In addition, in the subgroup analysis, the relationship between *H. pylori* detection methods and CIMT and PWV was different. In addition, in subgroup analysis according to different detection methods of *H. pylori*, the results showed that the relationship between *H. pylori* detection methods and CIMT was different, but there were significant correlations between the various methods and CIMT, which may be due to the different detection methods for *H. pylori* having different accuracies and precision.

To date, an increasing number of studies have proven a direct relationship between *H. pylori* infection and cardiovascular events. Eskandarian et al^[43]^ reported the impact of *H. pylori* infection on the risk of adverse cardiovascular events in patients with acute coronary syndrome and found that *H. pylori* infection has a significant impact on the prognosis of patients, which can lead to an increase in the risk of short-term adverse cardiovascular events. Recent studies have shown that patients with *H. pylori* infection have a 3- to 4-fold increased risk of CVD,^[44]^ including a 2-fold increase in the risk of myocardial infarction^[45]^ and a 1.1-fold increase in the risk of coronary heart disease.^[46]^ At the same time, some studies have shown that *H. pylori* infection can promote the development of carotid atherosclerosis. The authors followed up the patients for 3 years and found that compared with patients without *H. pylori*, the incidence of atherosclerosis from atherosclerotic to carotid atherosclerosis in young men with *H. pylori* infection was significantly increased.^[20]^

In addition, *H. pylori* infection is also a potential variable risk factor for the early prevention of CVD. A recent study,^[47]^ which analyzed a large database of 208,196 participants, showed that the incidence of coronary heart disease decreased after early eradication of *H. pylori*.

According to the above evidence and our meta-analysis results, *H. pylori* infection is an important risk factor for CVD. Because subclinical atherosclerosis is the early manifestation of CVD, early detection of subclinical atherosclerosis-related indicators in *H. pylori* patients can help prevent CVD. In this context, it provides a theoretical basis for the role of *H. pylori* eradication in the prevention of atherosclerosis in the future.

*H. pylori* infection and atherosclerosis are related to several potential factors. First, inflammation plays an important role in the occurrence and development of *H. pylori*. *H. pylori* toxin can stimulate host cells to produce inflammatory factors such as IL-1, IL-6, TNF-ɑ, and tumor necrosis factor, leading to chronic cellular inflammation and endothelial damage.^[48,49]^ In addition, *H. pylori* infection can lead to high-density lipoprotein cholesterol, low-density lipoprotein cholesterol, triglyceride, homocysteine, and lipid metabolism disorders.^[50,51]^ Third, chronic *H. pylori* infection may also be related to an increase in Von Willebrand Factor, which changes the lipid profile and leads to atherosclerosis.^[52,53]^*H. pylori* infection may be involved in the entire process of atherosclerosis.

There were some limitations in this meta-analysis. First, because the included studies were cross-sectional or case-control studies, the causal relationship could not be clarified. Second, the overall results showed significant heterogeneity, and subgroup analysis showed that patient age, sex, geographic location, study design, and different *H. pylori* testing methods may be sources of statistical heterogeneity. However, sensitivity analysis and publication bias showed that our results were stable. Third, the study population was mainly from Asia and Europe, lacking evidence from other regions. Fourth, most of the included studies used the stool antigen test, urea breath test, or serum IgG antibody test to diagnose *H. pylori* infection. Compared with endoscopic gastric biopsy, which is regarded as the gold standard, their accuracy in detecting *H. pylori* infection is limited. Fifth, the detection methods for CIMT or PWV were not entirely consistent.

## 4 Conclusions

In conclusion, our meta-analysis confirmed that *H. pylori* infection was associated with subclinical atherosclerosis. Early assessment and identification of subclinical atherosclerosis in patients with *H. pylori* infection may help formulate effective prevention and control strategies for cardiovascular events.

## Author contributions

**Conceptualization:** Xianghong Wang.

**Data curation:** Xianghong Wang, Kecheng Yao, Qian He.

**Formal analysis:** Donghua Jin, Baohua Ma, Xiulan Zou.

**Funding acquisition:** Xiulan Zou.

**Methodology:** Kecheng Yao.

**Resources:** Xianghong Wang, Donghua Jin, Baohua Ma, Xiulan Zou.

**Software:** Xianghong Wang.

**Supervision:** Xiulan Zou.

**Writing – original draft:** Xianghong Wang.

**Writing – review & editing:** Kecheng Yao, Qian He.
